# Heavy metals incorporated-alkali-activated waste glass binder and foamed glass: phase composition, physicomechanical performance, and reaction mechanism

**DOI:** 10.1038/s41598-026-65831-8

**Published:** 2026-08-07

**Authors:** Esraa K. Fayed, Fouad I. El-Hosiny, Hussein Al-kroom, Alaa A. Saleh, Ghada Bassioni, Mohammed Abd Elrahman, Hamdy A. Abdel-Gawwad

**Affiliations:** 1 Pyramids Higher Institution for Engineering and Technology, 6 October, Giza, 12451 Egypt; 2 Chemistry Department, Faculty of Science, Ain Shams University, Cairo, 12311 Egypt; 3 Department of Civil Engineering, School of Engineering, The University of Jordan, Amman, 11942 Jordan; 4 Composite and Hybrid Structures Department, TU Berlin, 13355 Berlin, Germany; 5 Raw Building Materials and Processing Technology Research Institute, Housing and Building National Research Center (HBRC), 87 El-Tahreer St., Dokki, Giza, Cairo, 12311 Egypt; 6 Chemistry Division, Faculty of Engineering, Ain Shams University, Cairo, Egypt; 7 Structural Engineering Department, Faculty of Engineering, Mansoura University, Mansoura, Egypt; 8 Department of Building Materials and Construction Chemistry, Institute of Civil Engineering, Technische Universität Berlin, Berlin, Germany

**Keywords:** Leaching rate, Minerals carbonate, Thermal conductivity, Phase composition, Heavy metals silicates, Chemistry, Engineering, Environmental sciences, Materials science

## Abstract

This study investigates the immobilization of hazardous witherite (BaCO₃) and zaratite (Ni₃(CO₃)(OH)₄·4 H₂O) as heavy-metal carbonates within alkali-activated systems subjected to thermal treatments. The objective was to compare alkali-activated waste-glass (WG) binders cured at 60 °C with foamed glass (FG) produced at 800 °C in terms of heavy-metal stabilization, phase evolution, physicomechanical performance, and thermal insulation. WG was replaced with 10 or 30 wt% carbonate; specimens were cured at 60 °C for 3 days and subsequently heated at 800 °C for 2 h. TG/DTG, XRD, FTIR, physicomechanical, thermal-conductivity, pore-structure, and acid-leaching analyses were performed. Witherite caused only a modest strength reduction, whereas 10 and 30 wt% zaratite reduced the compressive strength of cured binders by approximately 37% and 84%, respectively. Despite immobilization efficiencies exceeding 99%, Ba²⁺ and Ni²⁺ concentrations in the leachates remained above the permissible thresholds. At 800 °C, a 10 wt% dosage promoted foaming, producing approximately six-fold volume expansion and thermal conductivities of 0.09–0.12 W·m⁻¹·K⁻¹, whereas 30 wt% inhibited foaming. Unlike the cured binders, witherite and zaratite in FG transformed into acid-resistant barium and nickel silicates, principally sanbornite (BaSi₂O₅) and liebenbergite (Ni₂SiO₄). Consequently, Ba²⁺ and Ni²⁺ concentrations released from all FG samples were below the regulated limits and 300–500 times lower than those from the corresponding cured binders. This dual function of high-load heavy-metal carbonates as foaming constituents and precursors of stable silicates enables hazardous-metal stabilization and the production of lightweight thermal-insulation materials from waste glass.

**Supplementary Information:**

The online version contains supplementary material available at 10.1038/s41598-026-65831-8.

## Introduction

The secure management of hazardous waste streams generated by diverse industrial processes remains a critical focal point of environmental research, driven by the profound risks these materials pose to ecological integrity. Heavy metal-laden wastes, in particular, are classified as hazardous due to their acute toxicity and persistence. Furthermore, toxic metal accumulation within cementitious and concrete matrices may pose long-term environmental risks due to the possible release of hazardous elements under aggressive conditions^1–3^. Human exposure to these elements is linked to severe clinical pathologies, including dermatological hypersensitivity, renal and cardiovascular dysfunction, pulmonary fibrosis, and carcinogenesis^4,5^. Conventional landfilling of heavy metals presents substantial logistical and economic burdens, as it necessitates rigorous containment protocols that incur prohibitive costs. Therefore, incorporating heavy-metal-bearing wastes into construction materials represents a potentially sustainable management strategy, provided that the resulting products satisfy the relevant leaching limits under aggressive exposure conditions. Various remediation and stabilization approaches have been investigated for hazardous metal-containing wastes and polluted systems, including adsorption-based and chemically modified materials for enhanced metal ion removal from aqueous environments^6^.

Alkali-activation technology is considered one of the most effective approaches for the remediation of wastes rich in heavy metals^7,8^. Heavy metals can be retained in alkali-activated matrices through physical encapsulation, surface association, cation exchange, or chemical incorporation into reaction products^8–11^. Among these mechanisms, the formation of metal-silicate phases is generally considered more durable than physical retention alone, particularly under acidic exposure^8,9^. However, stabilization efficiency depends strongly on the metal species and dosage, precursor chemistry, reaction products, and exposure conditions^12–14^. Moreover, a high calculated immobilization percentage does not necessarily demonstrate environmental safety, because the absolute metal concentration in the leachate must also remain below the applicable regulatory limit.

The stabilization of nickel (Ni) and barium (Ba) is a global environmental priority, given their prevalence as byproducts in various industries^15–17^. Nevertheless, research specifically addressing their immobilization in alkali-activated materials remains limited compared with studies of Pb, Zn, Cd, and Cu. Few studies have focused on the immobilization of Ni- and Ba-rich wastes via the alkali-activation process^18–20^. Abdel-Gawwad et al.^21^ demonstrated that Ni-wastewater produced from the electroplating industry can be safely used as mixing water for alkali-activated slag (AAS). Leaching tests showed a high immobilization degree of these heavy metals through cationic exchange within the hydrotalcite phase or their localization at negative charges on alumina within the alkali-activated matrix. Although these findings demonstrate the capacity of alkali-activated matrices to retain Ni, the available investigations have predominantly considered dissolved Ni, industrial wastewater, or complex Ni-bearing residues. Consequently, the immobilization behavior of high contents of a defined nickel-carbonate mineral, such as zaratite, remains insufficiently understood.

Available studies on Ba immobilization have similarly focused mainly on slag-based matrices and relatively low dosages or highly reactive Ba sources. Incorporating nano-barium silicate into alkali-activated slag significantly improved its physical and mechanical properties, while Ba concentrations in the leachate remained below the permissible toxicity limit^22^. A similar trend was observed when nano-witherite was incorporated into alkali-activated slag^23^. Ba-rich industrial residues have also been investigated as components of alkali-activated binders^20,24^. These investigations suggest that Ba stabilization depends on the chemical form, particle size, incorporated dosage, and reactivity of the Ba-bearing material with the surrounding silicate matrix. However, their findings cannot be directly extended to high witherite loadings in waste-glass-based systems.

A critical limitation of previous Ni- and Ba-immobilization studies is their predominant focus on alkali-activated binders cured at ambient or moderately elevated temperatures. Under such conditions, acid-sensitive metal carbonates may remain partly unreacted, and their apparent stabilization may primarily result from physical encapsulation by the binder matrix. If this protective matrix deteriorates under acidic exposure, residual carbonate minerals may become exposed and release soluble metal species. This issue is particularly relevant to witherite (BaCO₃) and zaratite (Ni₃(CO₃)(OH)₄·4 H₂O), both of which can become unstable under acidic conditions. Therefore, it is necessary to determine whether alkali activation alone can safely stabilize high loadings of these minerals and whether subsequent thermal treatment can convert them into more acid-resistant phases.

It is well known that waste glass (WG) is a common precursor for fabricating alkali-activated binders^25–27^. The development of sustainable construction materials from industrial and municipal wastes has recently attracted increasing attention because of their environmental and functional benefits^28^. In addition to its use as an alkali-activated precursor, WG can be converted into foamed glass (FG), a lightweight cellular material with high porosity and low thermal conductivity. During heating, dehydration and dehydroxylation of the alkali-activated reaction products release gases, while the glass-rich matrix softens. Entrapment of the released gases within the softened glass produces the cellular structure characteristic of FG^29–33^.

Carbonate minerals may strongly influence this foaming process. Their thermal decomposition can release CO₂ and promote pore formation when gas evolution occurs within the glass-softening temperature range. Conversely, excessive carbonate replacement may dilute the silicate-rich matrix, change melt viscosity, or release gases before the glass softens sufficiently, thereby inhibiting foaming^34^. Witherite and zaratite may therefore perform a dual function: they may act as foaming constituents at suitable dosages, while their thermal-decomposition products may react with silica from WG to form Ba- and Ni-silicate phases. These silicate phases are expected to exhibit greater resistance to acidic attack than the original carbonate minerals. Nevertheless, the combined effects of witherite and zaratite on FG formation, pore structure, physicomechanical and thermal properties, phase transformation, and metal leachability have not been systematically investigated.

Accordingly, the present study compares the efficiency of alkali-activated WG binders and thermally produced FG in stabilizing exceptionally high contents of witherite and zaratite. WG was replaced with 10 or 30 wt% of either mineral to provide a rigorous assessment of the stabilization capacity of the two processing routes. The specimens were first cured at 60 °C for 3 days, after which corresponding samples were heated to 800 °C for 2 h to produce FG. The effects of mineral type, dosage, and thermal exposure on phase composition, reaction mechanisms, porosity, bulk density, pore structure, volume change, compressive strength, thermal conductivity, and Ba and Ni leachability were evaluated. The novelty of this work lies in elucidating the dual role of high-load heavy-metal carbonates as modifiers of the foaming process and precursors of acid-resistant Ba- and Ni-silicate phases. This comparison determines whether thermal foaming can simultaneously enhance heavy-metal stabilization and convert hazardous and glass wastes into lightweight thermal-insulation materials.

## Experimental procedures

### Materials resources

Waste glass (WG), witherite and zaratite minerals, and sodium carbonate (Na_2_CO_3_), sodium hydroxide (NaOH) are the main starting materials used for preparing foamed glass. The WG, obtained from waste laboratory chemical bottles at the Housing and Building National Research Center (HBRC, Egypt), was crushed and ground to yield WG powder. Witherite and zaratite were prepared by coprecipitation process via mixing barium chloride (BaCl_2_) and nickel chloride (NiCl_2_) with Na_2_CO_3_. The BaCl_2_ and NiCl2 with purity of 99.9% were sourced from LOBA chemical company (Mumbai, India). The details of the preparation process for the witherite and zaratite minerals are described in Sect. 2.2. NaOH with a purity of 97% and Na_2_CO_3_ with a purity of 99.9% were purchased from the Al-Nasr Pharmaceutical Chemicals Company (Cairo, Egypt).

Witherite and zaratite were not obtained commercially; instead, they were synthesized through a coprecipitation process. BaCl_2_ and NiCl_2_ solutions were individually mixed with Na_2_CO_3_ at an equimolar ratio. The dissolved heavy metal salts were precipitated as carbonates, according to the following simple exchange reactions:

1 $$BaCl2{\text{ }} + {\text{ }}Na2CO3{\text{ }}(aq.){\text{ }} \to {\text{ }}BaCO3 \downarrow {\text{ }} + {\text{ }}2NaCl$$

2 $$NiCl2{\text{ }} + {\text{ }}Na2CO3{\text{ }}(aq.){\text{ }} \to NiCO3 \downarrow {\text{ }} + {\text{ }}2NaCl$$

Following the completion of the precipitation process, the precipitates were washed several times to ensure the removal of dissolved sodium salt. They were then dried in an oven at 80 °C for 24 h, followed by gentle pulverization to produce a dried powder (cf. supplementary file, Figure S1). Although the above-mentioned exchange reaction confirms the formation of NiCO_3_, previous research^35,36^ has proven the formation of basic nickel carbonate (nickel hydroxy carbonate). This is investigated in detail in the following section.

### Materials characterization

**Fig. 1:**
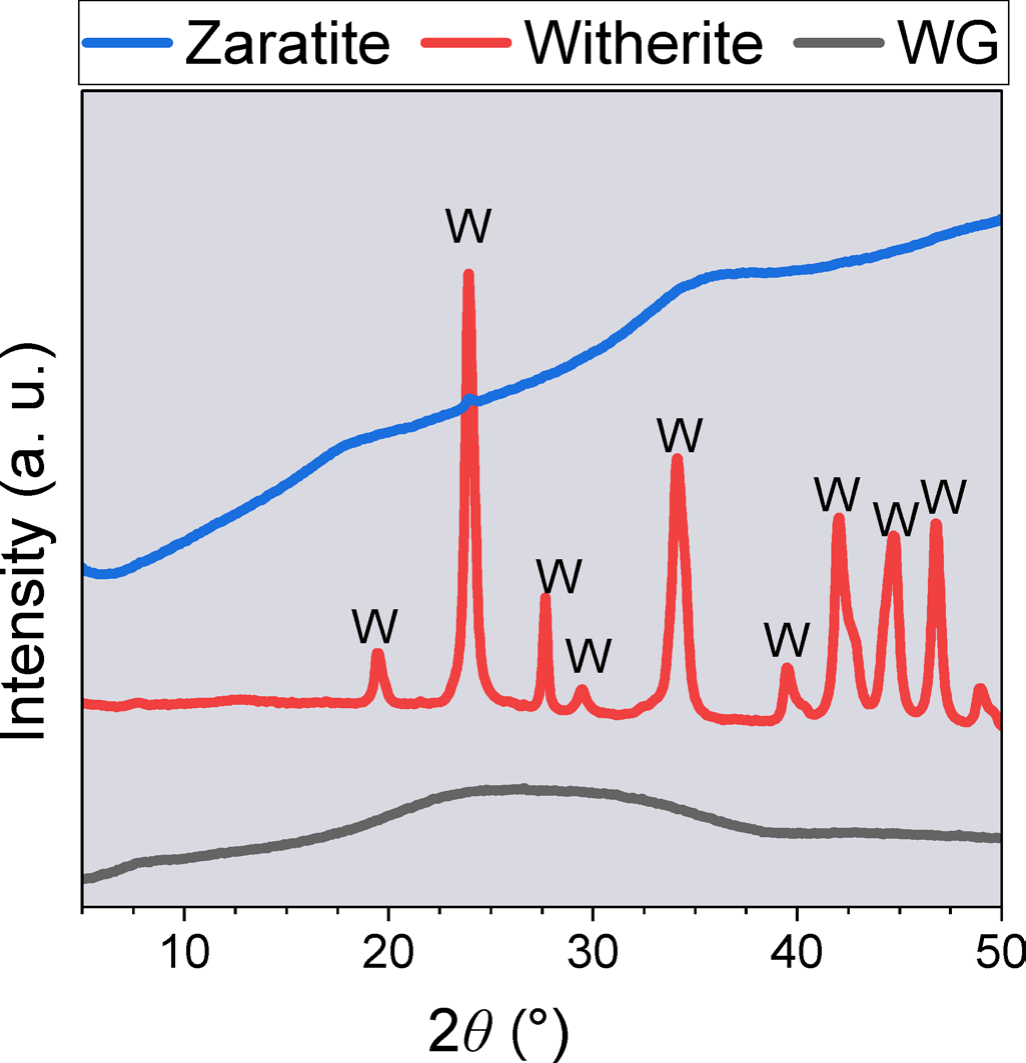
X-ray diffraction of waste glass (WG), witherite and zaratite (W = Witherite).

**Fig. 2:**
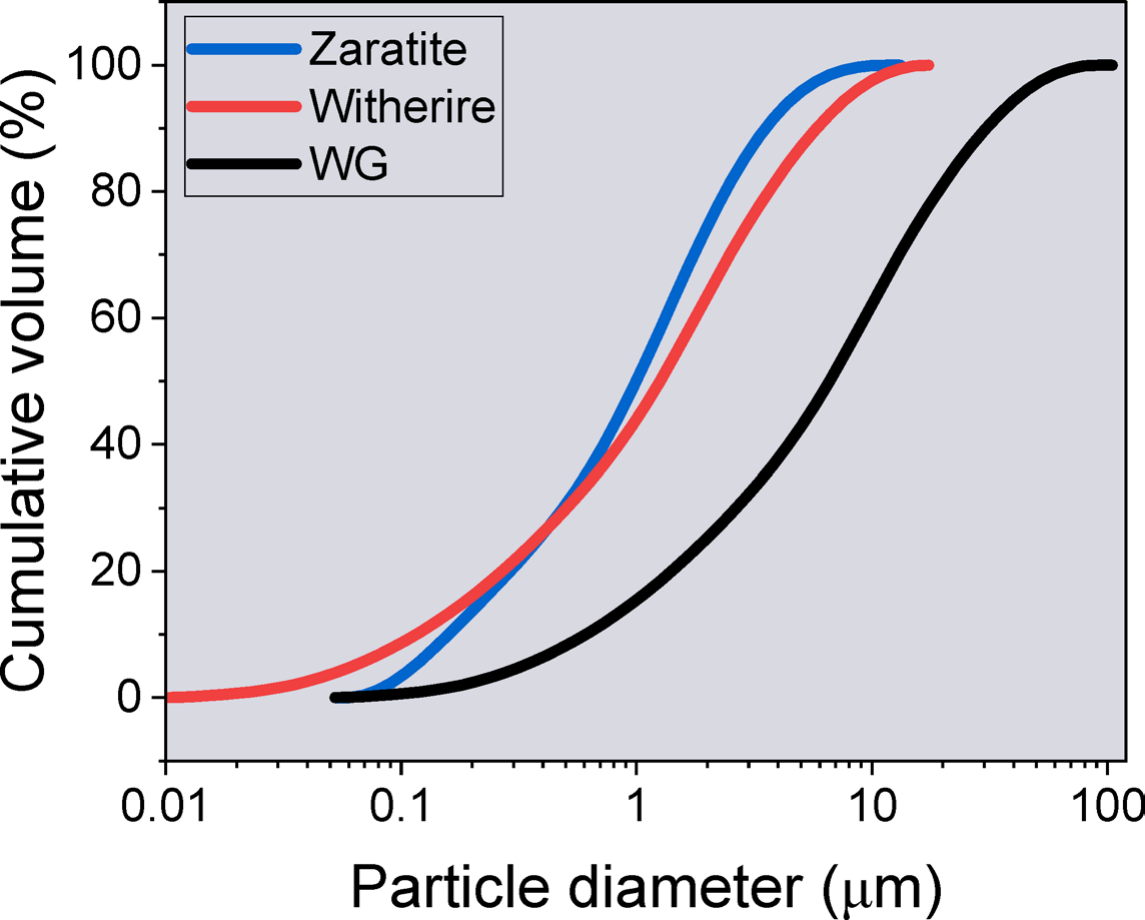
Particle size distribution of waste glass (WG), witherite and zaratite.

The oxide compositions of the starting materials, determined via X-ray fluorescence (XRF: Axios PW1400, PANalytical, Netherlands), are presented in Table 1. The analysis confirmed that the WG is primarily composed of silicon, calcium, and sodium oxides, with other oxides present in trace amounts. Although the stoichiometric weight loss of witherite is 22.30%, the synthesized sample exhibited a lower weight loss of 1.85 wt%. Conversely, the determined NiO content (57.62 wt%) is slightly lower than that of the standard zaratite mineral (59.57 wt%), indicating a purity of approximately 97%. The mineralogical compositions of the WG, witherite, and zaratite were determined using X-ray diffraction (XRD), as shown in Fig. 1. This analysis was conducted using an X’Pert PRO PW3040/60 (PANalytical, Netherlands) diffractometer equipped with a monochromatic Cu-Kα radiation source. The test was performed at 40 kV and 30 mA, with a scanning rate of 6 s/step, a resolution of 0.02°/step, and a 2θ range of 5–50°. WG and zaratite exhibit amorphous patterns, whereas witherite shows a crystalline structure with peaks corresponding to the witherite mineral. The specific gravity of the WG, witherite, and zaratite, measured using a pycnometer, is 2.97, 4.28, and 2.99, respectively. The particle size distribution (PSD), determined using a Mastersizer 2000 (Malvern Instruments, UK), is displayed in Fig. 2 for all starting materials.

**Table 1:** Oxide compositions of starting materials, as identified by x-ray fluorescence according to ASTM C114 (LOI was calculated after the exposure of samples to 950 °C).

Sample name	Oxides wt%											
	SiO_2_	CaO	MgO	Fe_2_O_3_	Al_2_O_3_	SO_3_	Cl	Na_2_O	K_2_O	NiO	BaO	LOI
WG	76.64	10.39	1.23	0.21	0.59	0.41	0.16	8.92	0.98	-	-	0.32
Witherite	-	-	-	-	-	0.1	0.26	0.31	-	-	97.39	1.85
Zaratite	-	-	-	-	-	-	0.15	0.18	-	57.62	-	42.03

**Fig. 3:**
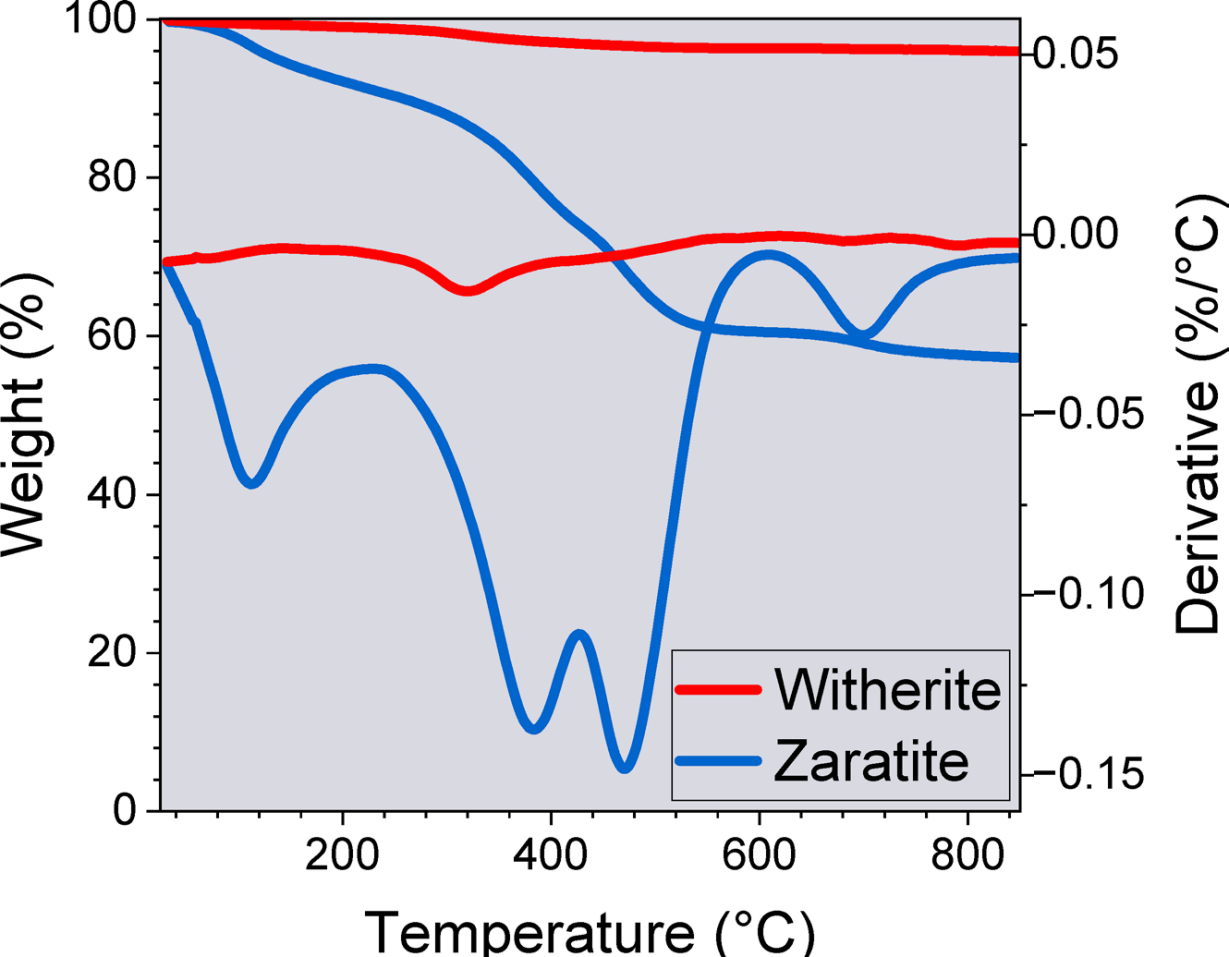
Thermogravimetric and derivative thermogravimetric analysis of witherite and zaratite minerals.

Since XRD results indicated an amorphous Ni-precipitate, thermogravimetric and derivative thermogravimetric (TG/DTG) analysis was employed using a TG 209 F3 Tarsus (NETZSCH Gerätebau GmbH, Germany) to confirm the formation of zaratite. The analysis was carried out by heating 10 mg of the sample in an alumina crucible up to 850 °C, at a heating rate of 5 °C/min under a nitrogen atmosphere. Three weight losses were detected, including the decomposition of combined water up to 200 °C, dehydroxylation from 200 to 450 °C, and decarbonation from 450 to 750 °C (Fig. 3). These peaks refer mainly to the formation of a nickel hydroxy carbonate hydrate mineral. The total weight loss (42.74 wt%) of the Ni-precipitate is comparable to that of the zaratite mineral (Ni_3_(CO_3_)(OH)_4_⋅4H_2_O), which is 41.43 wt%. The small deviation in the total weight loss of the precipitated samples is likely due to physically adsorbed water and contaminants, as shown in Table 1. Although witherite is easily detected by XRD analysis, no peak related to decarbonation weight loss was observed in this temperature window. This is mainly ascribed to the fact that the decarbonation of BaCO3 occurs at a higher temperature range of 1000–1300 °C^37,38^.

### Foamed waste glass preparation

Foamed glass (FG) was prepared in three stages. First, an alkali-activator solution was mixed with WG to produce a workable paste, which was then cast into 20 × 20 × 20 mm^3^ molds and covered with plastic sheets. The water-to-powder (W/P) ratio was kept constant at 0.35. NaOH was dissolved in the mixing water and then left for 24 h to cool to room temperature before being mixed with the solid precursors. Second, the samples were cured at 60 °C for 3 days. This temperature was selected to accelerate the hardening of the alkali-activated WG, owing to the low CaO content of the WG. The dried samples were heated to 800 °C at a rate of 20 °C/min and held at that temperature for 2 h. After the heating program was finalized, the samples were left to cool to room temperature. The NaOH concentration was adjusted to 4% by weight of the WG. The alkali-activator concentration and exposure temperature were selected based on previous studies^32,34^, as they represented the optimum conditions for producing high-performance FG. The influence of witherite (BaCO_3_) and zaratite (Ni_3_(CO_3_)(OH)_4_⋅4H_2_O) minerals on the performance of the FG was evaluated by replacing 10 wt% and 30 wt% of the WG with these minerals. The detailed mix proportions are provided in Table 2.

**Table 2:** Proportion of the investigated mixtures

Sample notation	WG	witherite	zaratite	NaOH	Na_2_Oeq	W/P
	wt%					0.35
G	100.0	-	-	5.3	4.0	
10BC	90.0	10.0				
30BC	70.0	30.0	-			
10NiC	90.0	-	10.0			
30NiC	70.0	-	30.0			

To distinguish between the samples cured at 60 °C and those exposed to 800 °C, the temperatures “60” and “800” were added to the mix codes. For instance, the cured G mixture was labeled as G-60, while the thermally exposed version was labeled as G-800, and so on.

### Physical and mechanical measurement

Bulk density, volume change, porosity, and thermal conductivity were determined to evaluate the physical performance of the produced foamed glass. Bulk density (kg/m^3^) was calculated according to ASTM C20^39^ by dividing the dry mass of the sample by its volume. The stability of the samples at elevated temperatures was measured by the difference in volume before and after thermal exposure. The relative volume is defined as the ratio of the sample volume after exposure to its volume before exposure. A ratio greater than one indicates sample expansion under elevated temperature, while a ratio less than one indicates shrinkage. High stability of the alkali-activated samples under elevated temperatures is achieved when the relative volume equals one. Finally, the porosity of the foamed glass was measured following the Archimedes method^39^.

The thermal conductivity (Tc) of the foamed samples was measured using a KD2 Pro thermal properties analyzer (Meter Group, Washington, USA) in accordance with ASTM D5334^40^. Because moisture within the samples can cause errors in thermal insulation measurements, Tc was measured immediately after thermal treatment at 800 °C. Alternatively, samples were dried at 100 °C for 24 h to remove any residual moisture. Tc was measured for three samples of each mixture, and the average values were reported.

The compressive strength of the alkali-activated samples (both cured at 60 °C and exposed to 800 °C) was measured using a German Brüf pressing machine with a maximum load of 175 kN. The test was performed on five hardened cubes per mixture to ensure the accuracy of the results. Relative strength (RS), which quantifies the change in compressive strength after exposure to 800 °C, was calculated by dividing the strength after thermal treatment by the strength before exposure. An RS value below one indicates a loss of strength, higher than one indicates a strength increase, and a value of one signifies no change.

### Barium and nickel leachability

A leaching test was performed on the nickel (Ni)- and barium (Ba)-containing samples to verify the safety of the produced FG for construction purposes. Additionally, the role of the alkali-activation process and thermal treatment in immobilizing Ni and Ba within the products was evaluated. This test was carried out following the Soluble Threshold Limit Concentration (STLC) procedures^41^.

The STLC test, an approved California method for characterizing hazardous waste, was conducted by suspending crushed samples of the alkali-activated material and FG (containing various dosages of witherite and zaratite) in a citric acid solution (0.1 M) at a liquid-to-solid ratio of 10:1. The suspension was agitated using a shaker at a rate of 60 rpm for 48 h. Subsequently, the leachate was separated from the solid materials via centrifugation at 4000 rpm. The resulting leachate was acidified prior to heavy metal measurement using an inductively coupled plasma atomic emission (ICP-AE) spectrometer (ULTIMA2, Horiba, Japan). This test was performed in triplicate for each mixture.

### Instrumental analyses

TG/DTG analysis was performed to determine the decomposition temperatures of the starting materials and alkali-activated mixtures. It was also used to identify the decarbonation temperatures of witherite and zaratite, associated with their transformation into other phases. XRD analysis was applied to determine the influence of witherite and zaratite minerals on the phase composition of the cured alkali-activated samples and FG. Fourier-transform infrared (FT-IR) spectroscopy was conducted using a Thermo Scientific Nicolet 6700 spectrometer to identify changes in functional groups resulting from the incorporation of carbonate minerals and thermal exposure. The analysis was carried out by scanning a wavenumber range of 400 to 4000 cm⁻¹ using KBr as a standard IR-transparent medium. Additionally, the pore distribution within the alkali-activated samples, both before and after thermal exposure, was investigated using a Stemi SV 11 stereomicroscope (Carl Zeiss, Jena, Germany).

## Results and discussion

### Phase identification

#### Thermal behavior

**Fig. 4:**
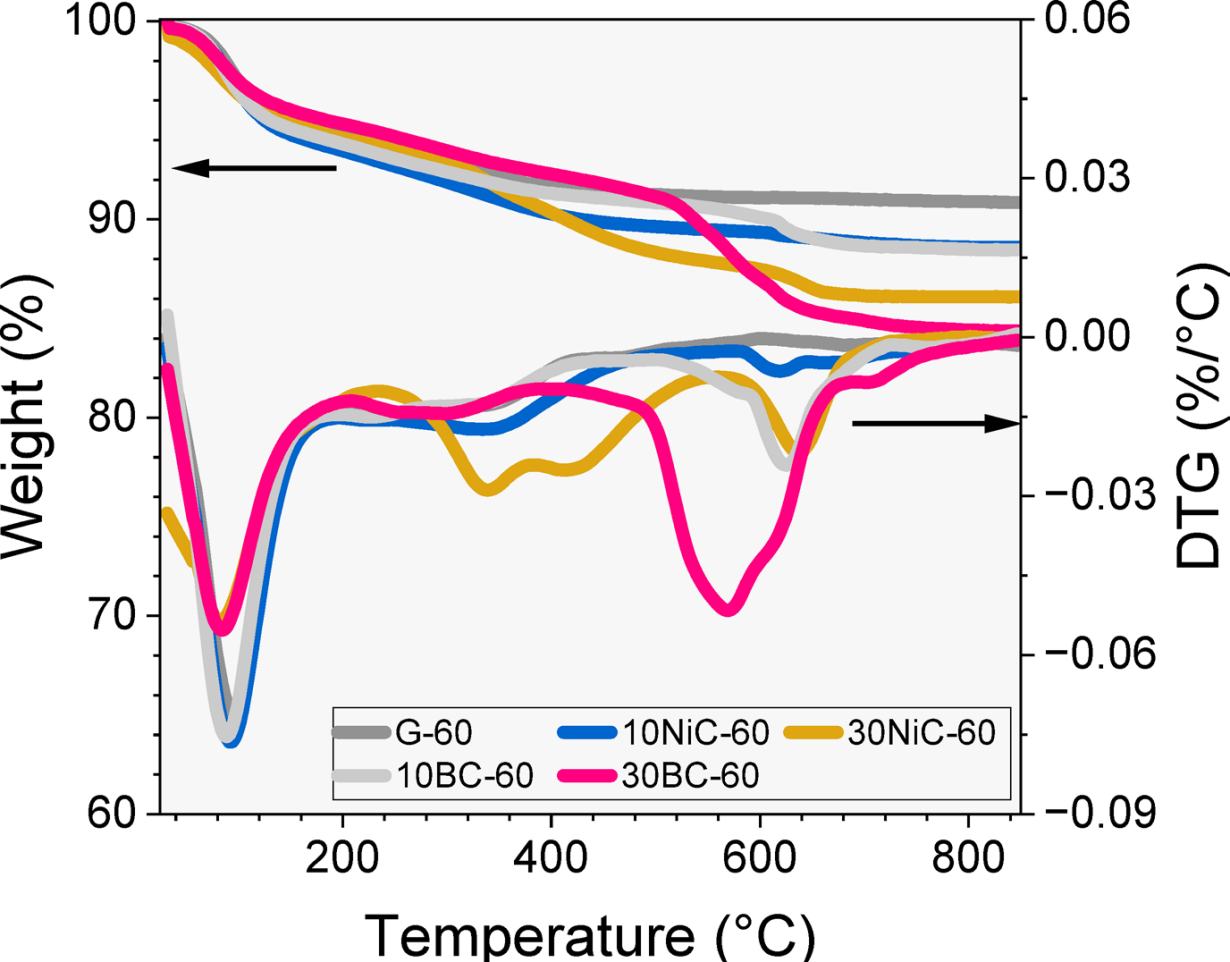
TG/DTG-curves of alkali-activated waste glass replaced with different contents of witherite and zaratite minerals cured at 60 °C for 3 days.

Figure 4 illustrates the impact of thermal exposure on the phase change and decomposition of alkali-activated WG, WG-witherite, and WG-zaratite mixtures. The TG/DTG curves exhibit distinct weight losses corresponding to the decomposition of various phases. For the control G-60 sample (without mineral additives), the weight loss occurring between 50 and 200 °C is attributed to the decomposition of combined water in sodium silicate hydrate (NSH) and physically adsorbed water within calcium sodium silicate hydrate (N(C)SH)^42–44^. An additional weight loss observed from 200 to 800 °C is associated with the dehydroxylation of Si-OH groups within the C(N)SH phase^45–47^.

Incorporating 10 wt% zaratite (10NiC-60) and witherite (10BC-60) increased the weight loss at 50–200 °C. The formation of nickel silicate hydrate (Ni-SH)^48^ is a likely reason for the increased weight loss in 10NiC-60 mixtures. In contrast, for the 10BC-60 mixture, the nucleating effect may drive the enhanced formation of hydration products. Thus, the increased weight loss observed when incorporating 10 wt% witherite and zaratite may be attributed to different mechanisms resulting from the varying thermodynamic stabilities of each carbonate, as discussed in Sect. 3.2. Furthermore, the addition of 10 wt% witherite and zaratite leads to the appearance of new peaks related to zaratite dehydroxylation and the decarbonation of both minerals within the 250–760 °C range. A further increase in the mineral content up to 30 wt% led to a decrease in weight loss at 50–200 °C. This is ascribed to the dilution of the silicate content, which negatively impacts the formation of silicate-based binders. Conversely, the higher dosage of both minerals increased weight loss between 250 and 760 °C, primarily due to the increased carbonate content.

Although pure witherite typically shows no substantial mass loss below 1000 °C in the present TG/DTG analysis (Fig. 3), the 10BC-60 and 30BC-60 mixtures exhibited distinct mass-losses between 500 and 760 °C. These losses are tentatively attributed to the decarbonation of witherite and other carbonate-bearing phases. The shift towards lower temperatures may reflect interactions with the alkali-activated matrix, including a possible fluxing effect of the activator^33,49^. However, carbonate decomposition temperatures can vary with composition, crystallinity, particle size, heating conditions, and interactions with surrounding phases. Moreover, overlapping decomposition reactions within this temperature range prevent the individual DTG peaks from being assigned conclusively to witherite based on thermal analysis alone^50,51^. The TG/DTG interpretation was therefore considered together with the XRD and FT-IR results obtained after heating the mixtures to 800 °C (Sect. 3.2 and 3.3). Collectively, these analyses indicate extensive decomposition of the carbonate-bearing and other thermally unstable phases by 800 °C, while the phase-specific assignments of the overlapping thermal events remain indicative rather than definitive.

#### XRD analysis

**Fig. 5:**
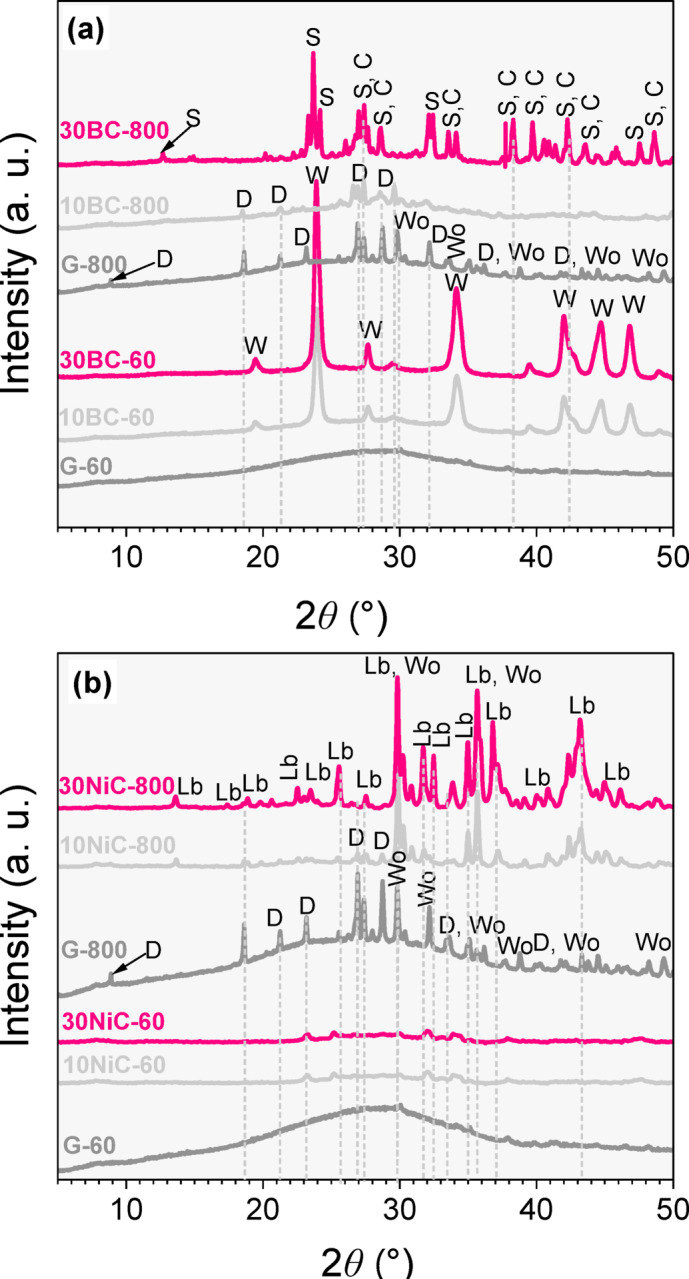
XRD-patterns of alkali-activated WG contains different (**a**) witherite and (**b**) zaratite contents cured at 60 °C for 3 days and exposed to 800 °C for 2 h.

The mineralogical compositions of alkali-activated binders cured at 60 °C (labeled as mixture-60) for 3 days and exposed to 800 °C (labeled as mixture-800) for 2 h are shown in Fig. 5a and b. The G-60 mixture exhibits an amorphous pattern with a hump between 20° and 40° 2θ (Figs. 5a, b). New peaks corresponding to the mineral witherite appeared within the patterns of the 10BC-60 and 30BC-60 mixtures (Fig. 5a). In contrast, no crystalline peaks were detected upon the incorporation of zaratite (10NiC-60 and 30NiC-60) (Fig. 5b), which is attributed to the inherently amorphous nature of the zaratite mineral (Fig. 1). The exposure of alkali-activated mixtures to 800 °C results in the formation of new minerals, the compositions of which rely primarily on the starting mixture. Wollastonite CaSiO_3_ and devitrite Na_2_Ca_3_Si_6_O_16_ are the main phases within the G-800 mixture (Figs. 5a, b). Exposure of the alkali-activated WG with 10 wt% witherite to 800 °C (10BC-800) hindered the formation of wollastonite and instead induced the formation of sanbornite BaSi_2_O_5_ and devitrite Na_2_Ca_3_Si_6_O_16_.

Increasing the witherite content to 30 wt% (30BC-800) enhanced sanbornite formation; however, instead of devitrite, peaks related to combeite Na_2_Ca_2_Si_3_O_9_ were identified (Fig. 5a). Despite these variations, there is clear evidence of the full decomposition of witherite within the alkali-activated mixtures at 800 °C. This confirms that the alkali-activator facilitates the decarbonation of witherite at temperatures lower than those required for its decomposition in the absence of an activator. This explanation aligns closely with the TG/DTG results of the alkali-activated mixtures (Fig. 4) and raw witherite (Fig. 3). Additionally, liebenbergite (Ni_2_SiO_4_) formation was induced by the incorporation of zaratite (Fig. 5b). This phase formed at the expense of devitrite Na_2_Ca_3_Si_6_O_16_ and wollastonite CaSiO_3_. In conclusion, the carbonate heavy metals within the alkali-activated mixtures transformed into heavy metal silicates at 800 °C. This occurred through the interaction of decomposed mineral carbonates with the reactive silica in the WG via the thermal mineral-induced formation phenomenon, as discussed in Sect. 3.3.

#### FT-IR analysis

**Fig. 6:**
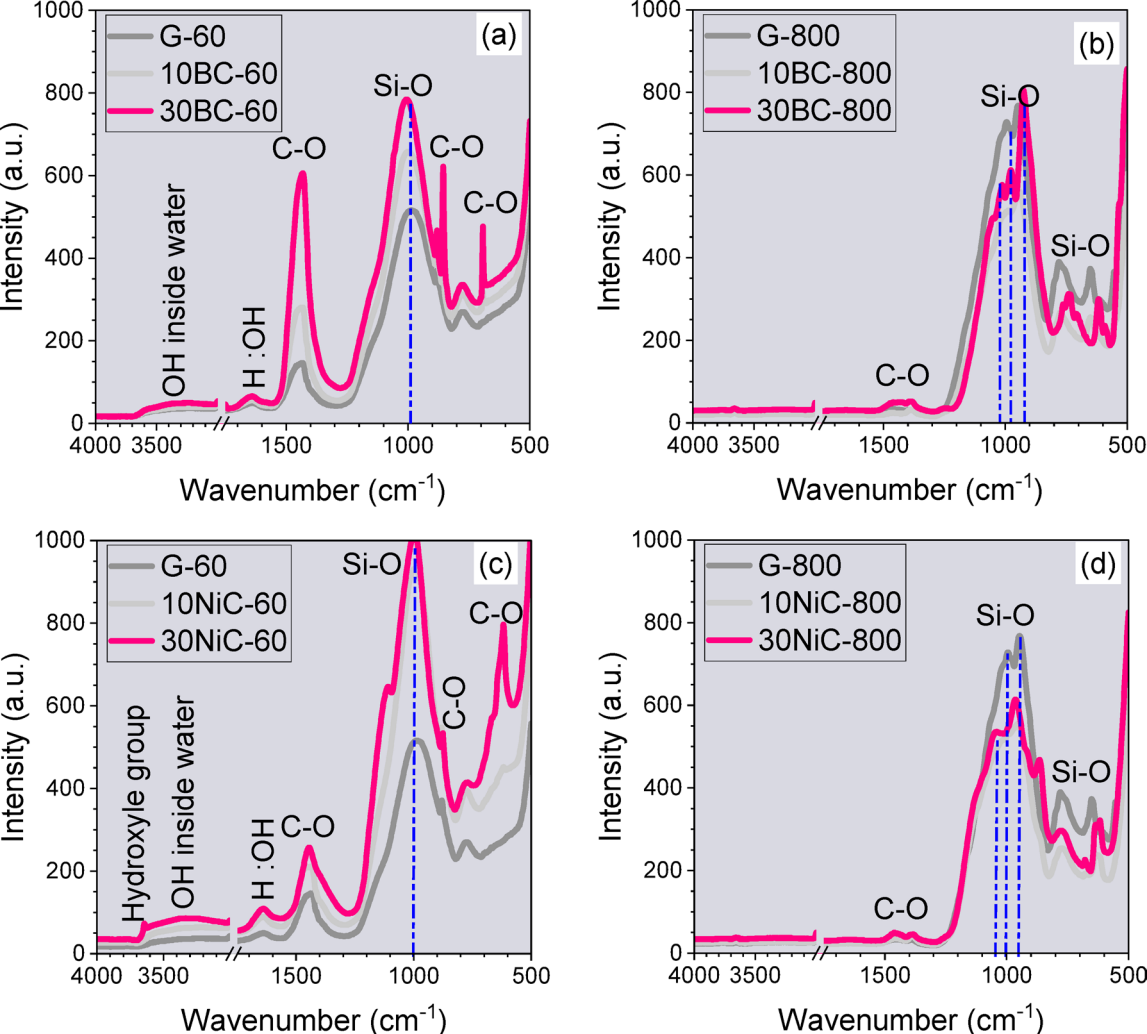
FT-IR-spectra of alkali-activated WG-witherite and WG-zaratite (**a**, **c**) cured at 60 °C for 3 days or (**b**, **d**) thermally exposed to 800 °C for 2 h.

FT-IR spectra reveal various bands corresponding to different functional groups, the composition of which depends primarily on the mixture design and exposure temperature (Fig. 6a–d). The cured alkali-activated waste glass (WG) (Fig. 6a, c) exhibits distinct bands at 726 cm⁻¹ and 989 cm⁻¹, attributed to Si–O bonds within the amorphous silica, NSH, and/or C(N)SH phases. Another band at 1430 cm⁻¹, affiliated with the CO_3_^2−^ group, confirms the presence of carbonate-containing phases within the activated binder, though these represent a minor component. The bands at 3493 cm⁻¹ and 1627 cm⁻¹ are primarily linked to chemically combined water within the NSH and C(N)SH phases^34^. The spectra of the cured alkali-activated WG-witherite and WG-zaratite mixtures show higher carbonate intensities compared to the WG-60 mixture (Fig. 6a, c). A shoulder detected at 3642 cm⁻¹ in the 30NiC-60 mixture suggests the presence of OH groups within the zaratite mineral^52^. Compared to the cured WG-witherite binders, the zaratite mixtures exhibit lower CO_3_^2−^ band intensity, specifically at 1430 cm⁻¹, which can be explained by the stoichiometrically lower carbonate content in the zaratite mineral.

Thermal exposure of the alkali-activated mixtures results in significant changes to the FT-IR spectra (Fig. 6b, d). A substantial reduction in bands relevant to carbonates and water was observed, consistent with the TG/DTG and XRD results (Figs. 4 and 5). The weak carbonate bands still present in the spectra of the thermally exposed mixtures likely originate from the atmospheric carbonation of unreacted alkaline earth metals (Ca, Na, and Ba), which may have occurred during sample handling after thermal exposure. Furthermore, various shoulders and a shift in the Si–O band at 989 cm⁻¹ toward both lower and higher wavenumbers were recorded. Additionally, new peaks in the 600–800 cm⁻¹ region were observed after exposing the mixtures to 800 °C. This is primarily ascribed to the formation of different silicate phases, such as wollastonite (CaSiO_3_), devitrite (Na_2_Ca_3_Si_6_O_16_), combeite (Na_2_Ca_2_Si_3_O_9_), sanbornite (BaSi_2_O_5_), and liebenbergite (Ni_2_SiO_4_), upon thermal treatment. These outcomes agree with the XRD results (Fig. 5).

### Physical and mechanical performance

**Fig. 7:**
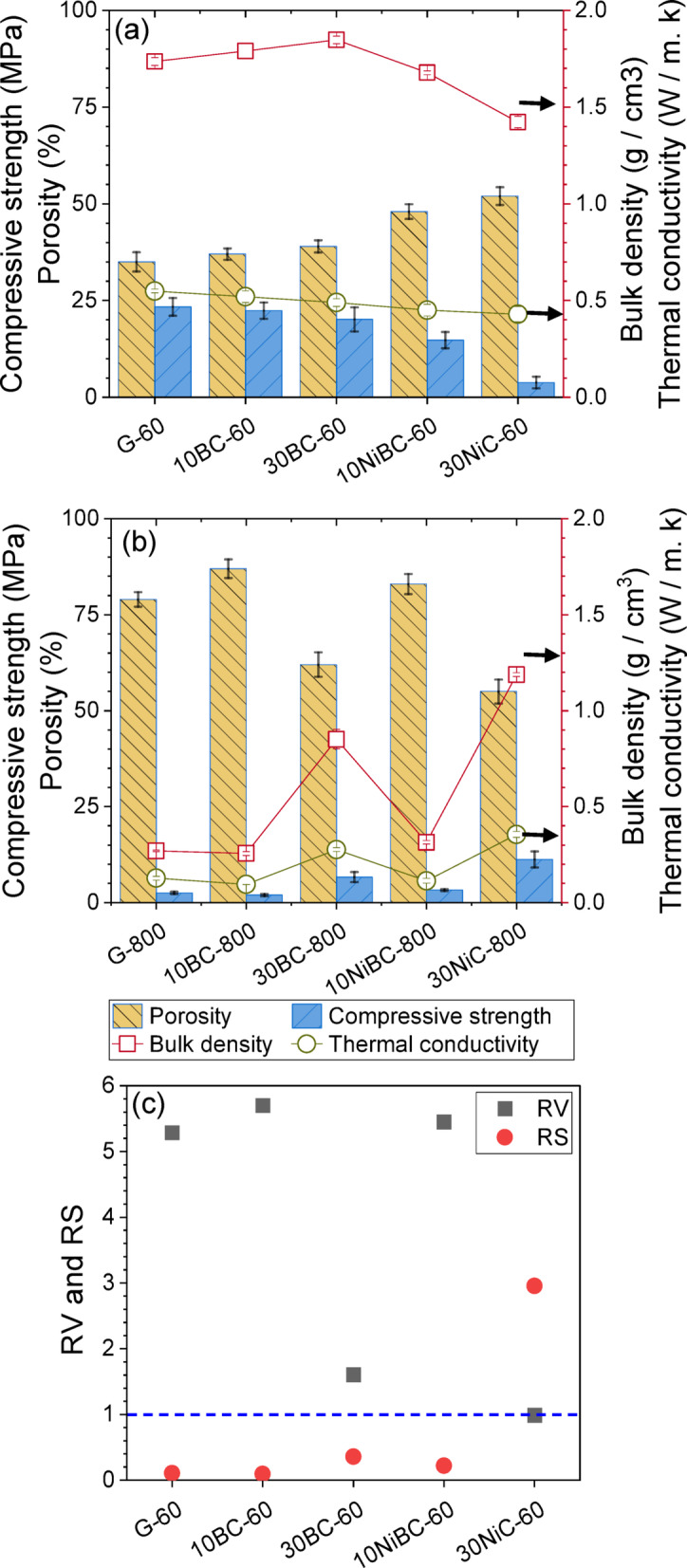
Compressive strength, bulk density, porosity and thermal conductivity of alkali-activated WG-minerals mixtures cured at (**a**) 60 °C for 3 days and (**b**) exposed to 800 °C for 2 h as well as (**c**) relative volume (RV) and relative strength (RS) of the thermally exposed mixtures. The presented data are the averages of three replicates for each mixture.

The role of witherite and zaratite minerals on the total porosity, bulk density, thermal conductivity, and compressive strength of the cured and thermally exposed mixtures is illustrated in Fig. 7a and b. The cured alkali-activated WG mixture exhibits a total porosity of 42%, a bulk density of 1.74 g/cm³, and a thermal conductivity of 0.55 W/m·K (Fig. 7a). Additionally, this hardened mixture records a compressive strength of approximately 23 MPa. Incorporating 10% zaratite/witherite has no significant effect on the compressive strength of the resulting hardened mixture. Conversely, replacing WG with 30 wt% witherite within the alkali-activated system reduces the compressive strength by approximately 10%. These outcomes are associated with slight changes in total porosity and thermal conductivity. Similar trends between porosity, density, and mechanical performance were also observed in eco-friendly cementitious composites containing recycled materials^53–55^. However, while the porosity increases slightly with the incorporation of witherite, the bulk density increases significantly (Fig. 7a). The difference in the specific gravity of WG (2.9) and witherite (4.28) is likely the reason for this finding.

In contrast, replacing WG with 10 and 30 wt% zaratite reduces the compressive strength of the resulting alkali-activated hardened mixtures by approximately 37% and 84%, respectively (Fig. 7a). This reduction is associated with a significant decrease in bulk density, total porosity, and thermal conductivity. The large variation in the effects of witherite and zaratite minerals on the performance of alkali-activated WG may originate from the tendency of each mineral to interact with the alkali-activator and, consequently, the dissolved silicate from the waste glass. Witherite is a barium carbonate known for its high thermodynamic stability and low reactivity within alkaline media^56^. The large ionic radius of barium (Ba) is the primary reason behind this stability and low reactivity; notably, it exhibits an atomic weight 2.3 times greater than that of nickel (Ni).

Zaratite reacts readily with NaOH within the system to yield nickel hydroxide Ni(OH)_2_) and sodium carbonate Na_2_CO_3_:

3 $$Ni_{3} CO_{3} (OH)_{4} \cdot 4H_{2} O{\text{ }} + {\text{ }}2NaOH{\text{ }} \to {\text{ }}3Ni\left( {OH} \right)_{2} \downarrow {\text{ }} + {\text{ }}Na_{2} CO_{3} {\text{ }} + {\text{ }}4H_{2} O$$

The formed Ni(OH)_2_ can then interact with the dissolved silica from the WG to produce Ni-SH. In contrast, the reaction between witherite and NaOH does not occur, primarily due to the high solubility of the potentially formed Ba(OH)_2_. Based on the Eq. 3, the interaction of zaratite with the alkaline solution and dissolved silica may promote the formation of Ni-containing silicate or Ni–S–H-like phases, which could contribute to Ni immobilization. However, the current XRD and spectroscopic results do not directly confirm the presence of a distinct Ni–S–H phase. The reduced Ni leaching may also result from precipitation, surface adsorption, incorporation into other reaction products, and physical encapsulation within the refined pore network. Therefore, Ni–S–H formation is proposed only as a plausible mechanism, and further experimental techniques and thermodynamic modeling are required to confirm this reaction.

Unlike the curing at 60 °C, the exposure of the alkali-activated WG, WG-witherite, and WG-zaratite mixtures to 800 °C resulted in a different trend (Fig. 7b). The total porosity of the WG-800 mixture increased from 35% to 79% after thermal exposure. This change was accompanied by a significant decrease in compressive strength and bulk density. Conversely, the thermal insulation of the resulting material significantly increased, with the thermal conductivity value decreasing from 0.55 to 0.13 W/m·K. The primary mechanism responsible for these variations in physical and mechanical performance is the foaming process induced by thermal exposure. This process is associated with the softening of the WG and the entrapment of decomposed water from the activation products. The resulting porous, lightweight material is commonly known as foam glass (FG). This reaction mechanism has been extensively discussed elsewhere^29,30^.

The 10BC-800 mixture exhibits higher porosity, lower bulk density, and lower compressive strength, accompanied by high thermal insulation. This indicates that a 10 wt% dosage of witherite enhances the foaming process, primarily due to the liberation of CO_2_ gas during witherite calcination. However, the foaming process is retarded with increasing witherite content; the 30BC-800 mixture shows lower porosity and higher bulk density, which is associated with a gain in compressive strength and an increase in thermal conductivity. A similar trend was observed in the WG-zaratite mixtures at the same exposure temperature of 800 °C. Nonetheless, these mixtures exhibit a lesser effect on the foaming rate compared to the WG-witherite mixtures, specifically at the 70 − 30 wt% ratio. From these outcomes, it can be concluded that 10 wt% of these carbonate minerals can act as a foaming agent, enhancing the bloating process of the softened glass at elevated temperatures. Conversely, the higher dosage exerts a retardation effect, suggesting a dilution of the silicate content, which is the fundamental basis for foamability under alkaline conditions and high temperatures. These findings align with previously published works addressing different carbonate minerals, such as calcite, magnesite, or carbonated concrete waste^34,57^.

The relative volume and relative strength are used to highlight changes in volume and compressive strength resulting from thermal exposure (Fig. 7c). Generally, the sample with the highest relative volume exhibits the lowest relative strength, and vice versa. An increase in relative volume indicates enhanced foamability and porosity, which is consistent with the results shown in Fig. 7b. The G-800 mixture records a volume expansion 5.3 times that of the original cured mixture (G-60). The highest volume expansion, 5.7 times, was recorded by the 10BC-800 mixture, which was accompanied by a decrease in relative strength. Incorporating 30 wt% witherite results in a significant decrease in volume change and a corresponding increase in relative strength. However, the relative strength remains below 1, indicating a net strength loss due to thermal exposure. The 10NiC-800 mixture exhibits relative volume and relative strength values comparable to those of the 10BC mixture. In contrast, the 30NiC-800 mixture shows no change in volume after thermal exposure, suggesting high thermal stability. This stability is associated with a significant increase in compressive strength, which is approximately three times greater than that of the cured sample (30NiC-60).

The changes in physical and mechanical properties are consistent with the phase transformations identified by XRD and FTIR (Figs. 5 and 6). For the control G-800 mixture, the formation of wollastonite and devitrite confirmed the thermal rearrangement and crystallization of the alkali-activated WG matrix. This transformation occurred concurrently with gas release from the hydrated binding phases, as supported by the reduction in the FTIR bands associated with water. Gas entrapment within the softened glass matrix produced a porous structure, explaining the increase in porosity and the corresponding reductions in bulk density, thermal conductivity, and compressive strength.

In the witherite-containing mixtures, the disappearance of witherite and carbonate-related FTIR bands after thermal exposure, together with the formation of sanbornite identified by XRD, confirmed the decarbonation of BaCO₃ and its reaction with silica. At 10 wt% witherite, CO₂ release promoted foaming, resulting in higher porosity and lower density, thermal conductivity, and compressive strength. Increasing the witherite content to 30 wt% enhanced sanbornite formation but reduced the available silicate-rich glass matrix and inhibited foaming. Consequently, the 30BC-800 mixture exhibited lower porosity and higher density, thermal conductivity, and compressive strength than the 10BC-800 mixture.

Similarly, the formation of liebenbergite in the zaratite-containing samples, supported by the development of new silicate-related FTIR shoulders and the reduction of carbonate and hydroxyl bands, confirmed the thermal transformation of zaratite. At 10 wt%, gas release contributed to pore formation and reduced density, thermal conductivity, and strength. At 30 wt%, however, much of the gas was released before sufficient softening of the WG matrix, while liebenbergite formation occurred at the expense of wollastonite and devitrite. This inhibited volume expansion and produced a denser and stronger thermally treated product. Therefore, the macroscopic performance of the investigated materials was governed by the combined effects of gas evolution, glass softening, and the composition-dependent formation of crystalline silicate phases.

### Microscopic investigation

**Fig. 8:**
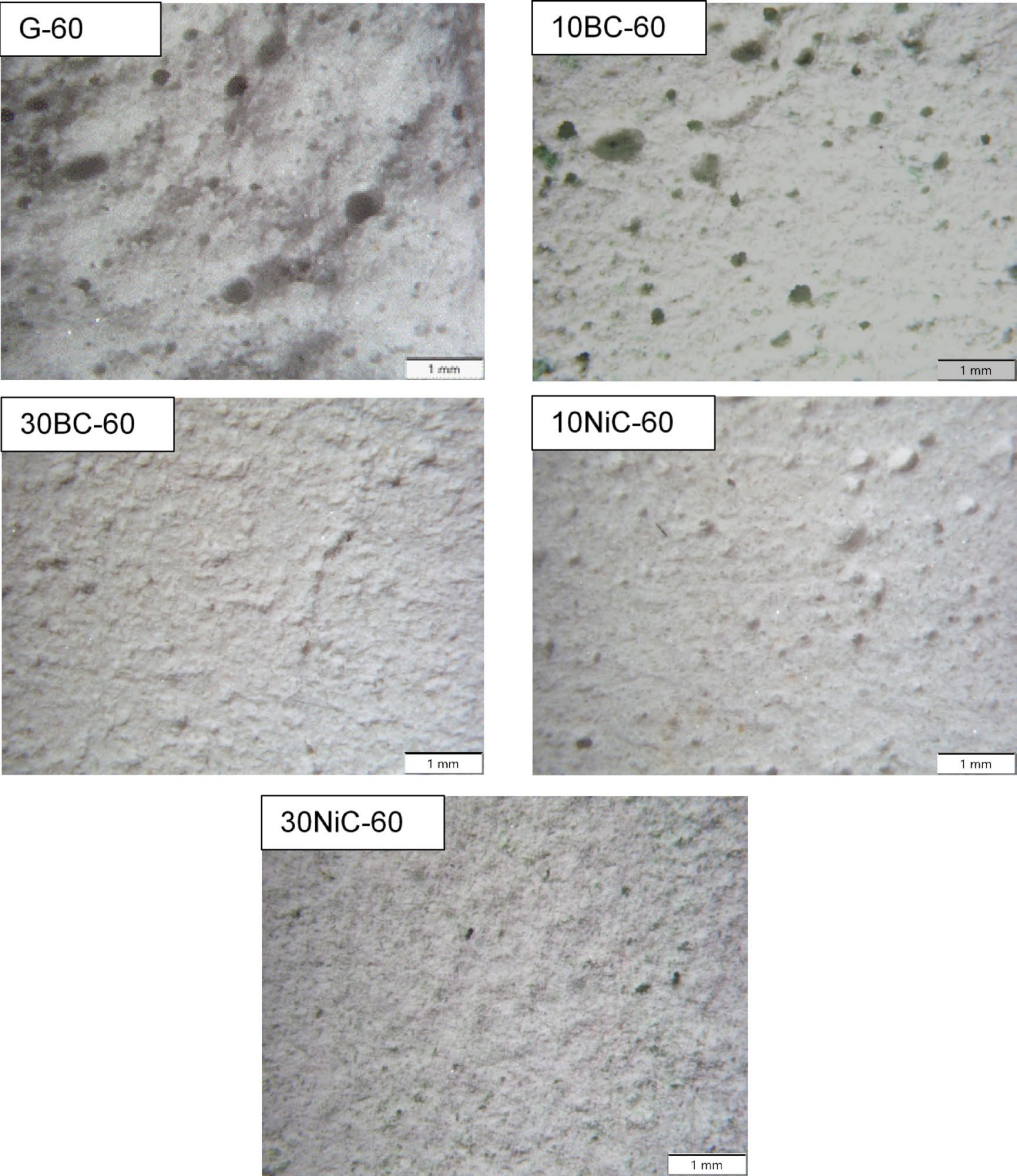
Stereomicroscopic photos of alkali-activated WG, WG-witherite, and WG-zaratite mixtures cured at 60 °C for 3 days.

**Fig. 9:**
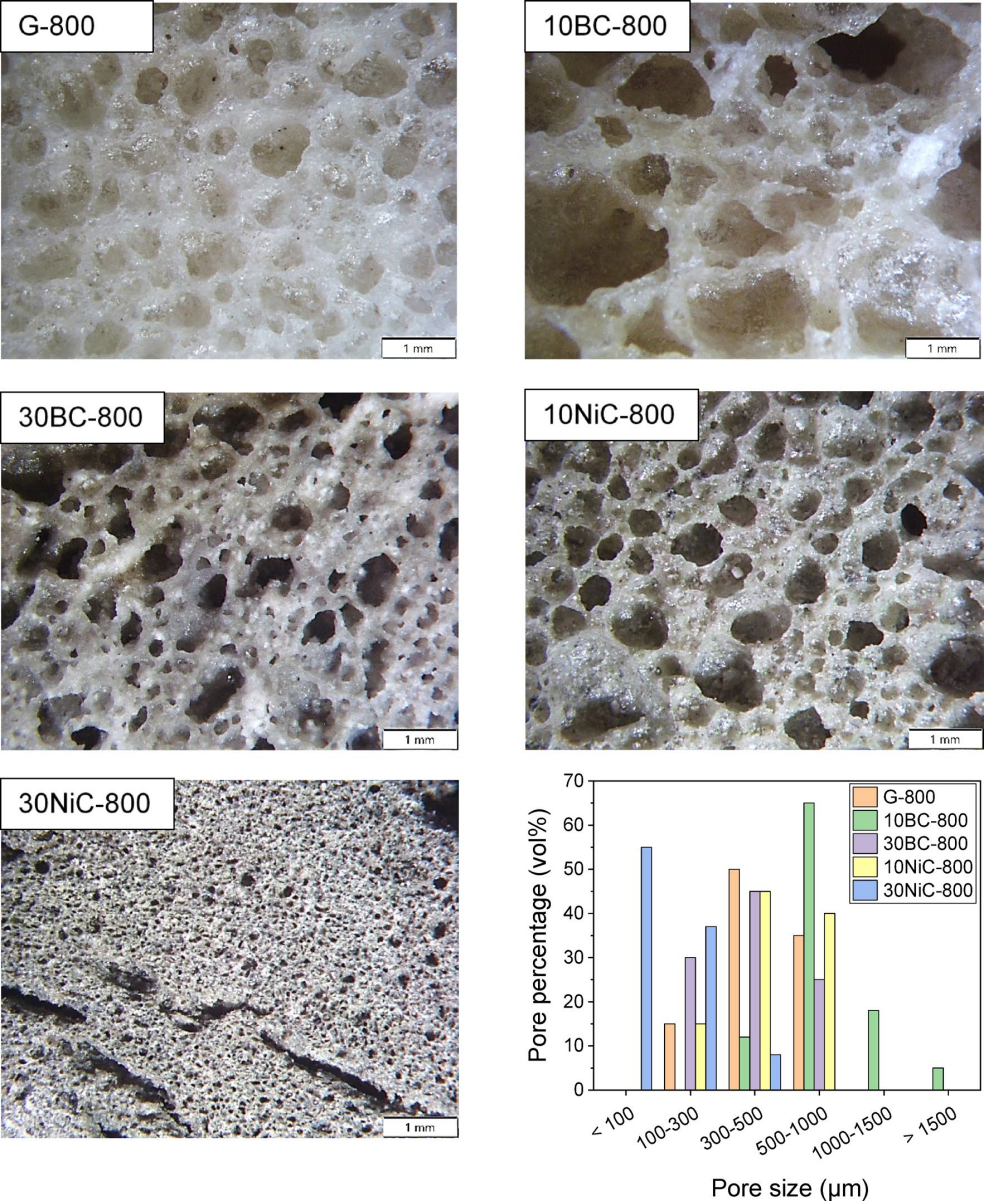
Stereomicroscopic photos as well as pore size distribution of alkali-activated WG, WG-witherite, and WG-zaratite mixtures exposed to 800 °C for 2 h.

Figures 8 and 9 display stereomicroscopic images of the alkali-activated WG samples cured at 60 °C and the FG samples, both with and without witherite and zaratite. These microscopic observations confirm the results shown in Fig. 7 by demonstrating the change in porosity after thermal exposure. Thermal exposure at 800 °C increased the porosity of the samples through the foaming process, resulting in a cellular microstructure.

The pore diameter of the produced FG depends primarily on the mixture composition (Fig. 8). In the G-800 sample, pore diameters ranging from 100 to 1000 μm were observed, with the majority of pores falling within the 300–1000 μm range. In contrast, the 10BC-800 sample exhibited larger pore sizes than those in the G-800 microstructure; this mixture recorded a mean pore diameter of 820 μm, significantly higher than the 540 μm observed for G-800. Furthermore, the 10BC-800 mixture reached a maximum pore diameter of 1650 μm, compared to 1150 μm for the G-800 sample. This indicates that incorporating 10 wt% of witherite not only increases total porosity (Fig. 7b) but also expands the pore diameter within the foamed glass (Fig. 8), confirming its role as a foaming agent that enhances the bloating process.

However, the sample containing 30 wt% witherite (30BC-800) showed the opposite effect, recording a lower mean pore size of 460 μm. While the WG-zaratite mixtures followed the same trend as the WG-witherite samples, they produced smaller pore diameters. Specifically, the 10NiC-800 and 30NiC-800 mixtures recorded mean pore diameters of 580 μm and 115 μm, respectively, compared to 820 μm and 460 μm for the 10BC-800 and 30BC-800 mixtures. These outcomes suggest that zaratite exerts a stronger retarding effect on the foaming process than witherite at a replacement level of 30 wt%. The decomposition of the majority of carbonates within zaratite occurs at temperatures significantly lower than 800 °C. This leads to the release of CO_2_ gas before the softening point of the WG, reducing the amount of entrapped gas inside the thermally exposed materials.

### Leaching and stabilization mechanism

**Fig. 10:**
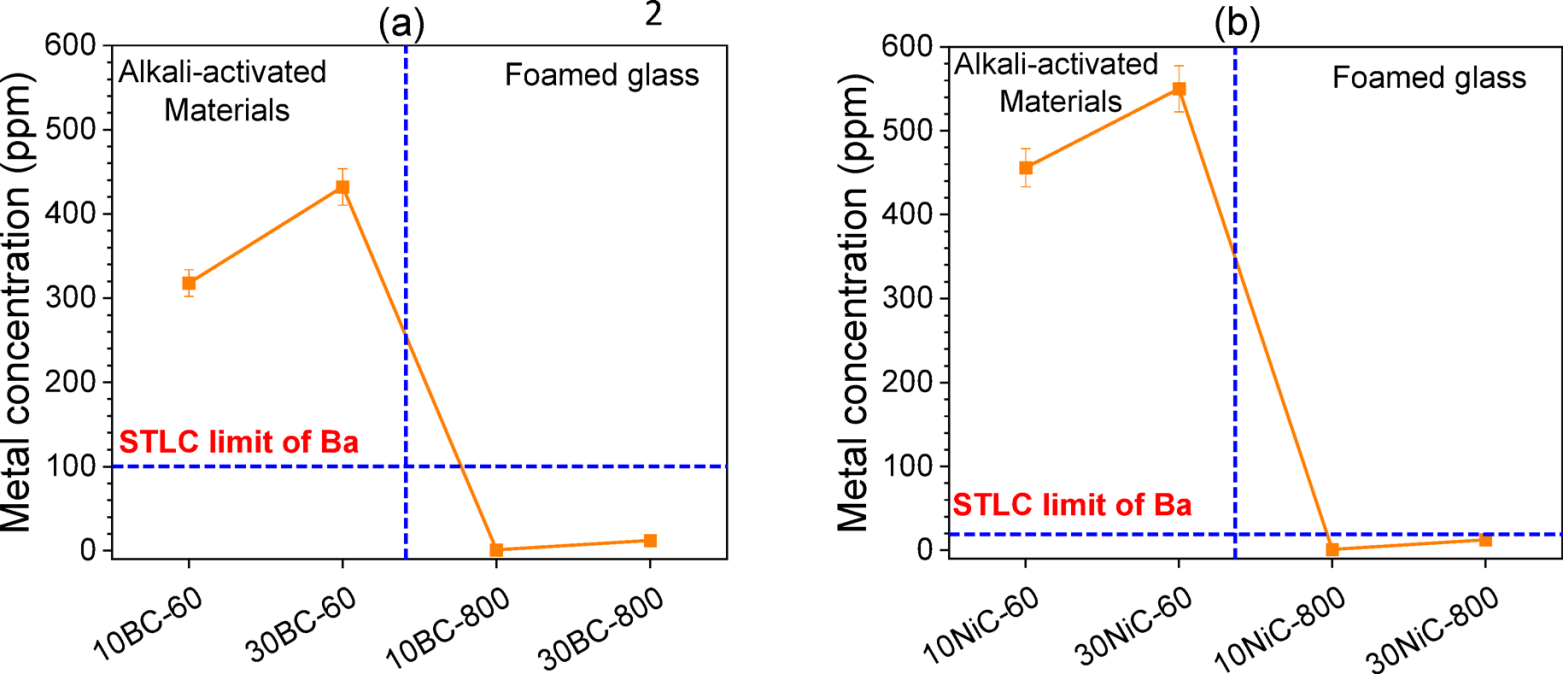
(**a**) Barium and (**b**) nickel metals concentration in the leachates of alkali-activated WG-witherite and WG-zaratite binders, respectively, and their relevant foamed glass (FG). (The content of Ba^2+^ inside starting WG-witherite powder are 6.9 × 10^4^ ppm and 20 × 10^4^ ppm. Whereas the Ni^2+^ concentration inside the WG-zaratite powder are 14 × 10^4^ ppm and 4.7 × 10^4^ ppm. The presented data are the averages of three replicates for each mixture.

It is well known that all carbonate minerals exhibit low stability under acidic conditions, as they can easily decarbonate to produce soluble salts. Witherite and zaratite, both heavy metal carbonates, transform from insoluble to soluble forms under acidic attack, posing serious environmental risks. Figure 10a, b illustrates the stability of these heavy metals (based on STLC leaching procedures) within the alkali-activated binders cured at 60 °C and the foam glass (FG) produced at 800 °C. Generally, for both the alkali-activated binders and FG samples, the concentration of dissolved heavy metals in the leachate increases with higher witherite and zaratite content. The cured alkali-activated binders substantially reduced Ba and Ni release compared with the untreated materials, demonstrating high immobilization efficiency relative to the reference materials. However, the final leachate concentrations remained above the applicable STLC limits (≤ 100 ppm for Ba and ≤ 20 ppm for Ni). Therefore, the observed reduction should not be interpreted as regulatory compliance or as unqualified evidence of high chemical durability. Instead, the results demonstrate effective but incomplete immobilization under the investigated conditions. The alkali-activated matrix was therefore unable to stabilize the high loading of metal-carbonate minerals sufficiently to meet the regulatory thresholds. Nevertheless, alkali activation may achieve more effective stabilization in acidic media at metal-carbonate contents below 10 wt%, consistent with previous studies^18,23,24^.

However, the objective of this study is to investigate the comparative efficiency of alkali-activation and the foaming process in stabilizing exceptionally high dosages of heavy metals, serving as a rigorous test of these approaches. Despite the high concentrations of dissolved minerals, the recorded Ba²⁺ concentration in the leachate of WG-witherite mixtures is lower than the Ni²⁺ concentration measured in the WG-zaratite leachate. This variation in leaching rates may be attributed to the difference in solubility between barium citrate and nickel citrate formed during the interaction with citric acid. Specifically, barium citrate exhibits lower solubility than nickel citrate^58^. At high witherite dosages, the potential precipitation of insoluble barium salts on the surface of the mineral may occur, thereby inhibiting further leachability. Nevertheless, all mixtures hardened at 60 °C pose serious environmental concerns and cannot be safely used as building materials, particularly in underground construction, because of the high toxicity associated with heavy-metal leaching.

The sintering of alkali-activated samples at 800 °C during the preparation of foam glass (FG) was found to exhibit the highest affinity for stabilizing witherite and zaratite. Notably, all FG samples yielded Ba²⁺ and Ni²⁺ concentrations below the STLC limits. A schematic diagram was developed to illustrate the effectiveness of both alkali-activation and thermal treatment processes in enhancing the stability of these heavy minerals. Furthermore, the reaction mechanisms underlying each process have been outlined.

Overall, As represented by recent work conducted by Tesovnik et al.^59^, heavy metal retention may arise from both chemical fixation and physical encapsulation, as pore refinement, lower connected porosity and permeability, and higher tortuosity restrict leachant access and ion transport. Metal mobility is also pH-dependent; therefore, the high alkalinity of alkali-activated materials may not provide optimal retention for all species. Controlled carbonation could mitigate leaching through pH regulation and microstructural modification. Recent work showed that post-curing carbonation reduced heavy-metal leaching while preserving performance, whereas premature carbonation increased microporosity and impaired strength. Accordingly, carbonation should be carefully optimized to balance leaching mitigation against possible phase destabilization and mechanical deterioration.

**Fig. 11:**
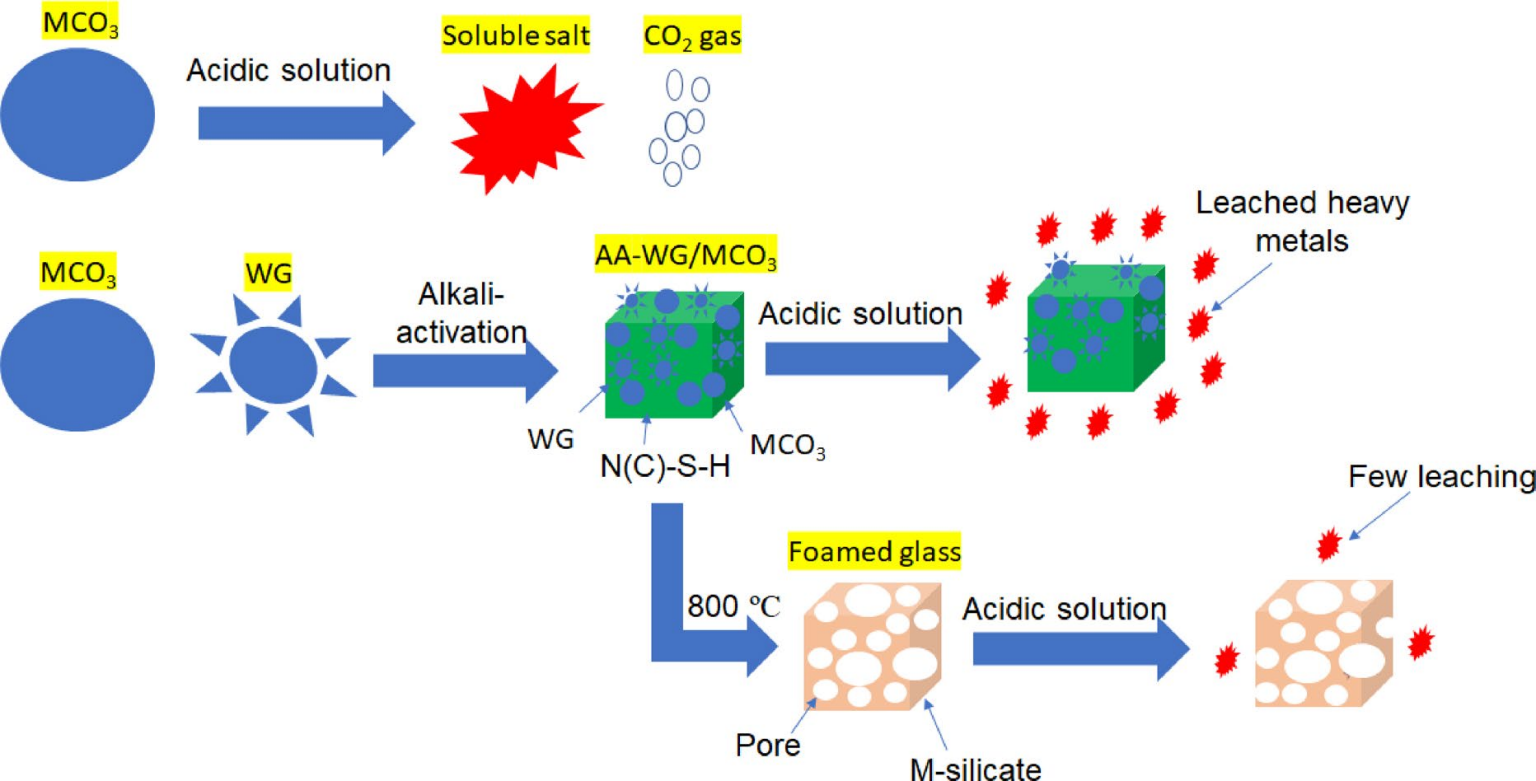
Schematic diagram shows the role of alkali-activation and thermal treatment in the stabilization of heavy metals inside the produced building products (M = Ni or Ba; N(C)SH = sodium calcium silicate hydrate; WG = waste glass).

As illustrated in Fig. 11, witherite and zaratite are susceptible to dissolution under acidic conditions, resulting in the release of soluble Ba and Ni species and CO₂. As discussed in Sect. 3.1.1 and 3.3, witherite behaved mainly as an unreacted filler in the alkali-activated matrix, whereas zaratite showed greater potential for reaction under alkaline conditions. In the alkali-activated binders, Ba and Ni retention was likely controlled by a combination of chemical fixation and physical encapsulation. The C-(N)-S-H-rich reaction products and unreacted waste-glass particles may physically surround the metal-bearing minerals, while pore refinement, reduced pore connectivity, lower permeability, and increased tortuosity restrict acid penetration and dissolved-ion transport. These combined effects explain the lower Ba and Ni release from the activated samples compared with the untreated powders. However, because the final leachate concentrations remained above the STLC limits, immobilization was effective but incomplete.

Thermal treatment produced a different and more effective stabilization mechanism. At 800 °C, the metal-carbonate phases decomposed to form Ba- and Ni-bearing products, which subsequently reacted with silicate species from the softened glass. XRD confirmed the formation of Ba- and Ni-bearing silicate phases (Fig. 5). Their incorporation into the thermally consolidated glass matrix, together with physical encapsulation and reduced accessibility to the leachant, increased resistance to acid attack relative to the original carbonate minerals. The superior immobilization achieved by thermal treatment can therefore be attributed to the combined effects of phase transformation, formation of more stable metal-bearing silicates, and encapsulation within the glass-rich matrix.

## Conclusions

This study compared alkali-activated waste-glass binders cured at 60 °C with foamed-glass products prepared at 800 °C for the stabilization of high witherite and zaratite contents. The principal conclusion is that alkali activation alone provided high apparent metal retention but was insufficient to ensure environmental compliance at the investigated carbonate loadings. In contrast, thermal foaming chemically transformed the acid-sensitive carbonates into more stable metal-silicate phases and reduced Ba and Ni release below the applicable limits.

- The effects of the carbonate minerals depended on their chemical reactivity and dosage. Witherite acted mainly as a relatively inert filler under alkaline curing conditions, whereas the higher reactivity of zaratite considerably reduced the strength of the cured binders. Therefore, the chemical form of the incorporated metal is a key factor controlling both binder development and stabilization performance.
- At 800 °C, witherite and zaratite performed a dual function. At 10 wt%, both minerals promoted foaming and produced lightweight materials with thermal conductivity values of 0.09–0.12 W·m⁻¹·K⁻¹. At 30 wt%, dilution of the silicate-rich glass matrix inhibited foaming and produced denser, stronger products. Accordingly, carbonate dosage provides a means of balancing thermal-insulation performance and mechanical strength.
- Thermal treatment converted witherite and zaratite into acid-resistant Ba- and Ni-silicate phases, principally sanbornite and liebenbergite, respectively. Metal release from the foamed-glass products was 300–500 times lower than that from the corresponding cured binders and remained below the applicable limits. This confirms that chemical conversion into stable silicates is more effective than physical encapsulation alone for stabilizing high carbonate loadings.

Overall, thermal foaming offers a promising route for combining hazardous-metal stabilization with the production of lightweight insulation materials from waste glass. However, the conclusions are limited to laboratory-synthesized witherite and zaratite, two replacement levels, one curing regime, one foaming temperature, and short-term citric-acid leaching conditions. Further studies should evaluate actual industrial wastes, intermediate carbonate contents, long-term leaching and durability under different environmental conditions, and optimization of the thermal process to reduce its energy demand.

## Supplementary Information

Below is the link to the electronic supplementary material.

Supplementary Material 1

## Data Availability

All data generated or analysed during this study are included in this published article.
