# Supplementary material for: Heavy metals incorporated-alkali-activated waste glass binder and foamed glass: phase composition, physicomechanical performance, and reaction mechanism
Source: Sci Rep. 2026 Aug 7;16:24504. doi: 10.1038/s41598-026-65831-8 (PMC13451210; doi:10.1038/s41598-026-65831-8)
Supplement: Supplementary file 1 [file 41598_2026_65831_MOESM1_ESM.docx]

Figure S1. Compressive strength of alkali-activated WG, WG-witherite and WG-zaratite cured at 60 C for 3 days.
